# Protein Preparations as Ingredients for the Enrichment of Non-Fermented Milks

**DOI:** 10.3390/foods11131817

**Published:** 2022-06-21

**Authors:** Katarzyna Kiełczewska, Aneta Dąbrowska, Marika Magdalena Bielecka, Bogdan Dec, Maria Baranowska, Justyna Ziajka, Yang Zhennai, Justyna Żulewska

**Affiliations:** 1 Department of Dairy Science and Quality Management, Faculty of Food Science, University of Warmia and Mazury, Oczapowskiego 7, 10-719 Olsztyn, Poland; kaka@uwm.edu.pl (K.K.); anetazj@uwm.edu.pl (A.D.); decbog@uwm.edu.pl (B.D.); mbb@uwm.edu.pl (M.B.); justyna.ziajka@uwm.edu.pl (J.Z.); jzulewska@uwm.edu.pl (J.Ż.); 2 Beijing Advanced Innovation Center for Food Nutrition and Human Health, Beijing Engineering and Technology Research Center of Food Additives, Beijing Technology and Business University, Beijing 100048, China; yangzhennai@th.btbu.edu.cn

**Keywords:** non-fermented milks, milk protein, amino acid profile, calcium, phosphorus, viscosity, heat stability, color, sensory analysis

## Abstract

Milk enriched with functional ingredients of milk proteins delivers health and nutritional benefits, and it can be particularly recommended to consumers with increased protein requirements. The aim of this study was to evaluate the applicability of casein and serum protein preparations obtained by membrane filtration in the laboratory as additives to non-fermented milks, as compared with commercial protein, preparations (whey protein isolate or concentrate and casein concentrate). The addition of protein preparations increased the pH, viscosity and heat stability of non-fermented milks. Milks enriched with whey proteins were characterized by a higher content of valine and isoleucine and a lower content of leucine, lysine and arginine. Addition of casein or whey protein concentrate decreased the phosphorus content and increased the calcium content of milk, but only in the products enriched with casein or whey protein concentrate. Color saturation was higher in products fortified with protein preparations obtained in the laboratory and commercial whey protein concentrate. Milk enriched with whey protein isolate, followed by milk serum protein concentrate, received the highest scores in the sensory evaluation. The presented results make a valuable contribution to the production of milks enriched with various protein fractions. The study proposes the possibility of production of protein preparations and milks enhanced with protein preparations, which can be implemented in industrial dairy plants.

## 1. Introduction

Milk and dairy products are rich sources of valuable nutrients such as protein, fat, lactose and minerals, including calcium [1,2]. Consumers are increasingly aware of the health benefits associated with dairy products and incorporate these foods, in particular fermented milks, in their regular diets [3]. Dairy products are abundant in wholesome proteins, which are a rich source of exogenous amino acids [1,2,4] and precursors of biologically active peptides [5] that promote metabolic processes and contribute to human health [2,6,7].

Cow’s milk proteins are composed of two fractions: casein that accounts for around 80% of total proteins, and serum proteins which make up around 20% of total proteins. Casein occurs in the form of αs1-, αs2-, β-, γ- and κ-casein, which carry calcium, phosphorus, iron, zinc and copper ions. Milk contains casein micelles with a size of 100–200 nm. Whey proteins include lactalbumins, lactoglobulins, immunoglobulins, enzymes and mineral-binding proteins such as lactoferrin [8]. The high nutritional value of milk proteins can be attributed to the high content and bioavailability of essential amino acids. Milk proteins are more bioavailable and digestible than plant-based proteins because they do not contain antinutritional factors [9]. Nutritional value and bioactive properties of milk proteins are also important considerations in the production of non-fermented milks. Dairy products are classified as functional foods if they contain ingredients which, in addition to providing nutrients, beneficially modulate specific functions in the body. Milk enriched with functional ingredients of milk proteins can be addressed to specific groups of consumers with higher protein requirements, including pregnant and lactating women, older persons at risk of sarcopenia, athletes and convalescents [10].

In the European Union, milk proteins and protein preparations are not regarded as food additives and can be used in the production of clean label foods [11]. These functional ingredients are applied in the food processing industry in view of high solubility, dispersibility, water binding, foaming, whipping, emulsification, gelation and buffering capacity [12], and are therefore used in an increasing number of food applications to modify the heat stability, viscosity, texture and sensory properties of products [13].

Heat stability is a particularly important consideration in the production of milk with increased protein content and/or modified protein composition. Colloidal stability is determined by many factors, including active acidity and the ionic environment of casein micelles, content of milk proteins, ratio of casein to whey proteins, dispersion of casein micelles and the proportion of κ-casein, hydration, calcium and phosphorus balance, and the proportions of colloidal and soluble forms of calcium and phosphorus [14]. Destabilization is observed when protein concentration and the activity of calcium ions in the solution increase [15], whereas a decrease in the calcium content of high-protein milk improves heat stability [16]. The result of the colloidal stability of milk is its viscosity and sensory properties.

Milk proteins are increasingly obtained by membrane filtration which enables the isolation of native proteins such as micellar casein and undenatured whey proteins. In comparison with other isolation techniques (such as isoelectric precipitation or enzymatic hydrolysis), the filtration process is more effective in preserving the functional properties of the isolated milk proteins [17].

The aim of this study was to evaluate the applicability of casein and whey protein preparations, obtained from skim milk by membrane filtration, as additives to non-fermented milks, as compared with commercial protein preparations, based on the technological properties (including heat stability and viscosity, which play an important role in the production of non-fermented milks with modified protein content and composition), nutritional value (based on calcium and phosphorus content and the composition of exogenous amino acids) and sensory attributes of the end products, which influence consumer acceptance.

## 2. Materials and Methods

### 2.1. Materials

**Figure 1:**
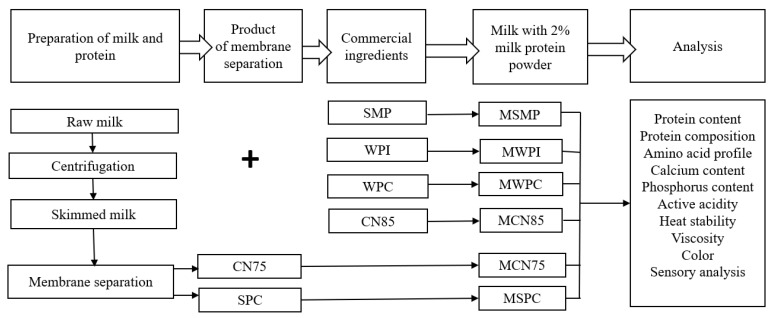
Experimental design. SMP—skim milk powder; WPI—whey protein isolate; WPC—whey protein concentrate; CN85—micellar casein preparation with 85% protein content; CN75—micellar casein concentrate with 75% protein content; SPC—serum protein concentrate; MSMP—milk with skim milk powder, control sample; MCN75—milk with micellar casein concentrate with 75% protein content; MCN85—milk with micellar casein concentrate with 85% protein content; MSPC—milk with serum protein; MWPI—milk with whey protein isolate; MWPC—milk with whey protein concentrate.

The analyzed material was skim milk powder (SMP) (Mlekpol, Grajewo, Poland). Raw milk with 3.44% protein content, 4.2% fat content and 4.86% lactose content was heated to 45 °C and centrifuged in the LWG20 separator (Spomasz, Gniezno, Poland). Skim milk was enriched with SMP (control sample) or protein preparations at 2% (experimental samples). The following commercial protein preparations were used in the study: micellar casein preparation with 85% protein content (CN85) (Inleit Ingredients, Curtis-A Coruña, Spain), whey protein isolate with 91% protein content (WPI), whey protein concentrate with 60% protein content (WPC) (Superior Ltd., Olsztyn, Poland), as well as protein preparations obtained from skim milk by membrane filtration: micellar casein concentrate containing 75% of protein (CN75) and serum protein concentrate containing 67% of protein (SPC) (Figure 1).

The prepared samples with the addition of SMP and protein preparations were defined as non-fermented milks, and they were divided into the following groups for analysis: milk with the addition of skim milk powder (MSMP) and milk with the addition of protein preparations (MCN75, MSPC, MCN85, MWPI, MWPC). Skim milk with the addition of SMP was the control (Figure 1). The experiment was conducted in duplicate.

### 2.2. Separation of Casein and Serum Protein Preparations from Skim Milk

#### 2.2.1. Production of Micellar Casein Concentrate

Raw whole milk was obtained from a dairy farm and centrifugally separated into raw cream and raw skim milk in a dairy plant operated by the University of Warmia and Mazury in Olsztyn. Raw skim milk was pasteurized with a plate heat exchanger at 72 °C with a holding time of 16 s. Pasteurized skim milk was microfiltered three times (three-stage process) at 50 °C using a pilot-scale ceramic microfiltration system in continuous bleed-and-feed mode with a concentration factor (CF) of 3× to produce microfiltration (MF) retentate and MF permeate. On the same day, water diafiltration was conducted twice to complete the three-stage process [18].

#### 2.2.2. Microfiltration Process

A pilot-scale graded permeability microfiltration system equipped with 0.1-μm ceramic Membralox membranes (model EP1940GL0.1μAGP1020, alumina, Pall Corp., East Hills, NY, USA) was used to microfilter skim milk. During the MF process, retentate and permeate were collected continuously in separate stainless steel tanks and immediately cooled to 4 °C. Typical inlet pressure and retentate outlet pressure were 2.5 and 1.4 bar, respectively, when the permeate valve was fully opened (atmospheric pressure on the permeate side). At the end of the MF run, all retentates and permeates from the processing run were combined and sampled. The MF experiment was replicated three times.

#### 2.2.3. Diafiltration Processes

The MF retentate was diafiltered (DF) twice to obtain native micellar casein concentrate with a reduced content of serum proteins (SP). The MF retentate (from the first stage) diluted by weight with reverse osmosis (RO) water to back to the initial concentration was the feed for the first diafiltration. Retentate and water were combined before heating to 50 °C and processed in the MF system under the same operating conditions as those applied in the first stage. The third stage (second DF) of the three-stage process was identical to the second stage (first DF), where the retentate from the second stage diluted with RO water was used as the feed. All retentates and permeates were collected, cooled, combined and sampled [18].

Serum proteins removed from milk by MF were further purified and concentrated by ultrafiltration to produce serum protein concentrate (SPC).

#### 2.2.4. SPC Produced in the Pilot Plant

The MF permeate and the DF permeate from the first diafiltration were weighed, combined, heated to 50 °C and ultrafiltered (UF stage 1 at CF 5×) and diafiltered (DF stage 2 at CF 8×) to produce 60% liquid SPC. The final UF retentate (liquid SPC) was cooled, held overnight at 4 °C and spray-dried.

The feed for the ultrafiltration process was fractionated using a pilot-scale UF system in continuous bleed-and-feed recirculation mode. The UF system was equipped with a semipermeable poly-ethersulfone (PES) spiral wound membrane (model: 3838 HFK-131, Koch Separation Solutions, Wilmington, MA, USA) with nominal pore size of 10,000 Da. The conditions and parameters of UF processing were as follows: feed temperature—around 50 °C; retentate inlet pressure—around 4.2 bar for UF and around 3.8 bar for DF with no back pressure on the permeate side.

Before DF, RO water was added to the UF retentate in an amount corresponding to the permeate obtained during UF. When the desired CF was achieved, permeate and retentate streams were directed to cooling and storage tanks. The final UF retentate and permeate were combined at the end of the process.

#### 2.2.5. Spray Drying

The final CN75 and SPC retentates were spray-dried using a standard Production Minor Spray Dryer (Niro Atomizer, Soeborg, Denmark). Inlet temperature was 180 °C and outlet temperature was 80 °C. The dried product was collected and packaged in hermetically sealed plastic bags.

#### 2.2.6. Protein Content

The protein content of raw milk and non-fermented milks with high protein content was determined with the use of the MilkoScan™ FT2 analyzer (Foss, Hillerød, Denmark).

#### 2.2.7. Reducing-Sodium Dodecyl Sulphate Polyacrylamide Gel Electrophoresis (SDS-PAGE)

The protein profile of the analyzed samples was determined by SDS-PAGE electrophoresis. Before the analysis, milk samples were defatted in the Eppendorf FT15 laboratory centrifuge. Skim milk samples were diluted in Laemmli 2× concentrate S3401 sample buffer (Sigma Aldrich, St. Louis, MO, USA). Samples were boiled for 5 min at 95 °C to denature proteins, and they were cooled to room temperature. They were centrifuged (13,000 rpm, 21 °C, 15 min) and loaded into wells on 4–20% Mini-PROTEAN^®^ TGX™ gel, in 15-well plates (Bio-Rad Laboratories Inc., Hercules, CA, USA). The Precision Plus Protein Dual Color Standards (Bio-Rad Laboratories Inc., Hercules, CA, USA) for proteins with a molecular weight of 10–250 kDa were used as the reference bands. Gel was placed in the buffer chamber, and a solution of 10× tris/glycine/SDS running buffer was added (Sigma Aldrich, St. Louis, MO, USA). The gel was initially run at 80 V, and the voltage was gradually increased to 120–150 V as the samples were absorbed by the gel. Gels were run in the BIO-RAD Mini-protean II cell apparatus (Bio-Rad Laboratories Inc., Hercules, CA, USA). Gels were stained and destained with Coomassie Brilliant Blue [19]. Gels were read in the CCD LumiBis imaging system (DNR Bio-Imaging Systems, Modi’in-Maccabim-Re’ut, Israel). The protein content of the samples was determined by densitometry with the TotalLab Quant 1.0 program (TotalLab, Gosforth, UK).

#### 2.2.8. Amino Acid Profile

The content of exogenous amino acids, threonine (Thr), valine (Val), isoleucine (Ile), leucine (Leu), phenylalanine (Phe), histidine (His), lysine (Lys) and arginine (Arg), as well as tyrosine (Tyr) as a conditionally essential amino acid, was determined in milk samples subjected to acid hydrolysis. Acid hydrolysis was conducted in the HB 016 thermo-block (Ingos Ltd., Prague, Czech Republic) in boiling 6 N HCl (110 °C) for 24 h, according to the official reference procedure [20]. Cooled milk samples were transferred to a 1 mL Eppendorf reaction vessel and evaporated in the RVO 400 vacuum rotary evaporator (Ingos Ltd., Prague, Czech Republic) to dryness at 60 °C.

The amino acid profile of the hydrolysates was determined in the AAA 500 automatic amino acid analyzer (Ingos Ltd., Prague, Czech Republic) with an ion exchange column (Indos Ltd., Prague, Czech Republic) and post-column ninhydrin derivatization. The spectrophotometric analysis was conducted at a wavelength of 440 nm for proline and 570 nm for the remaining amino acids. The ninhydrin reagent (Ingos Ltd., Prague, Czech Republic) and the buffer system (Ingos Ltd., Prague, Czech Republic) were prepared according to the manufacturer’s recommendations. The flow rate was 0.3 mL·min^−1^ for buffers and 0.2 mL·min^−1^ for the ninhydrin reagent. Each hydrolysate was analyzed in duplicate. Amino acids were identified by comparison with the amino acid reference chart (Indos Ltd., Prague, Czech Republic).

#### 2.2.9. Calcium and Phosphorus Content

Calcium and phosphorus content was determined in samples subjected to dry mineralization. The samples were mineralized in a muffle furnace at a temperature of 550 °C until the achievement of light-colored ash without traces of carbon [21]. Calcium content was determined by atomic absorption spectrometry in an air-acetylene flame with the use of the iCE 3000 Series Atomic Absorption Spectrometer (Thermo-Scientific, Hemel Hempstead, UK) equipped with a deuterium lamp for background correction and cathode lamps for each element, according to standard methods [22] at a wavelength of 422.7 nm. The content of phosphorus was measured colori-metrically with ammonium molybdate, sodium sulfate and hydroquinone (Merck, Darmstadt, Germany) in the Cary 60 UV-VIS spectrophotometer (Agilent, Santa Clara, Canada) at a wavelength of 610 nm [23].

#### 2.2.10. Active Acidity and Heat Stability

The pH of non-fermented milks was determined with the CPC 505 pH meter (Elmetron, Zabrze, Poland) equipped with an Inode electrode and calibrated with standard solutions with pH 4.0 and 7.0 (Merck, Darmstadt, Germany). Heat stability was measured as the time of heat coagulation at a temperature of 140 °C in an oil bath (TewesBis, Barczewo, Poland) [24].

#### 2.2.11. Viscosity

The shear stress of non-fermented milks was analyzed with the Rheolab QC rotational rheometer (Anton Paar GmbH, Graz, Austria) in the DG42-SN20765 measuring system, at a temperature of 20 °C and a shear rate of 1–100 s^−1^. The resulting flow curves revealed a linear relationship between changes in shear stress as a function of the shear rate. Viscosity was expressed by the slope of a straight line as the tangent at the point where the flow curve meets the *X*-axis.

#### 2.2.12. Color

The color of milk was analyzed with the CM-3500d spectrophotometer (Konica Minolta Sensing Inc., Osaka, Japan) which measures color transmittance and reflection. Measurements were performed with d/8 geometry, 8 mm aperture size, 10° observer angle and D65 illuminant. Before analysis, the device was calibrated against a white calibration plate (CM A120) and a black calibration plate (CM A124). A representative sample was prepared at room temperature immediately before analysis and placed in a CM A-128 Petri dish (h = 25 mm, Ø = 34 mm) [25].

Color lightness was calculated in the CIE LAB color space and expressed by component L* (L* = 0 for black and L* = 100 for white color). Chromaticity of the light source was described with components a* (−a* = greenness and + a* = redness) and b* (−b* = blueness and +b* = yellowness). The results were used to calculate saturation C* with formula C* = (a*^2^ + b*^2^)^0.5^. The total difference in color between the white standard and non-fermented milks was calculated with the use of formula ΔE = (ΔL^2^ + Δa^2^ + Δb^2^)^0.5^ [26].

#### 2.2.13. Sensory Analysis

The sensory analysis of non-fermented milks enriched with protein preparations was conducted in a sensory laboratory with the use of the profiling method described by EN ISO 13299:2016-05E [27], on a five-point descriptive scale. The analysis involved eight panelists who had been trained to evaluate dairy products and whose sensory sensitivity had been validated according to EN ISO 8586:2014-03 [28]. The panelists used definition cards and score cards to evaluate the intensity of 38 sensory attributes, where 1 point denoted the absence of the analyzed attribute, and 5 points denoted very high intensity of the analyzed attribute.

### 2.3. Statistical Analysis

Significant differences in heat stability, viscosity, calcium and phosphorus content, color parameters, amino acids, and sensory attributes were determined by one-way ANOVA and Fisher’s LSD test. All results were processed in Statistica 13.5 PL software (Statsoft 2017, Krakow, Poland) at *p* ≤ 0.05.

## 3. Results and Discussion

### 3.1. Proximate Composition

**Figure 2:**
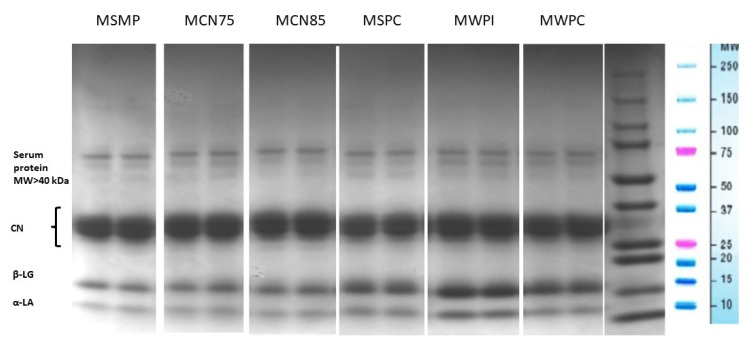
SDS-PAGE electrophoretogram of milk samples. MSMP—milk with skim milk powder, control sample; MCN75—milk with micellar casein concentrate with 75% protein content; MCN85—milk with micellar casein concentrate with 85% protein content; MSPC—milk with serum protein; MWPI—milk with whey protein isolate; MWPC—milk with whey protein concentrate.

**Figure 3:**
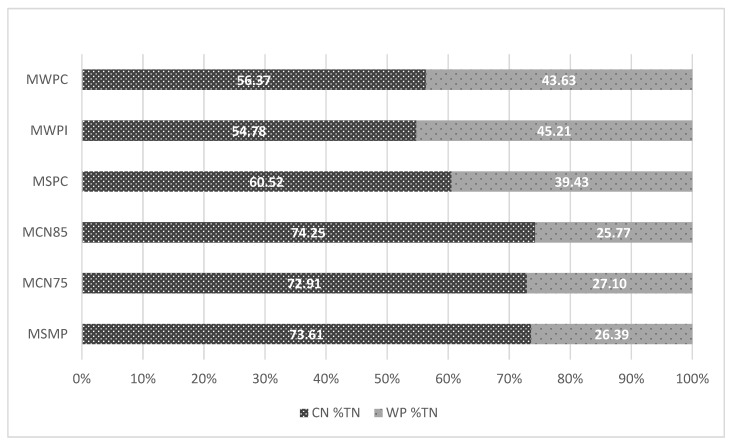
Average content of casein and whey proteins in a densitometric analysis of electrophoretograms (*n* = 2). CN—casein; WP—whey protein; TN—total nitrogen; MSMP—milk with skim milk powder, control sample; MCN75—milk with micellar casein concentrate with 75% protein content; MCN85—milk with micellar casein concentrate with 85% protein content; MSPC—milk with serum protein; MWPI—milk with whey protein isolate; MWPC—milk with whey protein concentrate.

The protein content of non-fermented milks is influenced by the type of added protein preparations. Protein content was highest (5.89%) in MWPI, and it was determined at 5.52% in MCN75, 5.64% in MCN85, 5.40% in MSPC, and 5.31% in MWPC. The proportions of casein (CN) and whey proteins in the total protein (TP) content of non-fermented milks were determined by the type of protein preparation (Figure 2 and Figure 3).

The application of CN75 and CN85 did not induce changes in CN’s share of TP relative to the product with the addition of SMP. Similar results were noted by Barłowska et al. [29] in raw milk, where CN accounted for 74.78% of TP on average. In milk enriched with whey proteins, α-lactalbumin and β-lactoglobulin accounted for up to 45% of TP, and their content was highest in milk with the addition of WPI (Figure 3).

During milk filtration, some protein molecules cause internal membrane fouling due to their chemical binding mechanisms and interactions with the membrane (protein molecules are adsorbed onto the membrane, especially on relatively hydrophobic polymers such as poly-sulfone), as well as protein–protein interactions which lead to the formation of agglomerates. Both phenomena can change the membrane’s sieving characteristics relative to the constituent proteins [30].

The extent to which milk proteins contribute to membrane fouling process is determined by differences in the size distribution and other characteristics of protein molecules, such as charge and hydrophobicity [31]. Research has demonstrated that serum proteins are more prone to adsorption on the membrane than casein, where β-lactoglobulin contributes most to membrane fouling, whereas α-LA strongly binds Ca^2+^ and promotes calcium-mediated salt bridging between α-LA and the membrane. The main serum proteins (α-LA, β-LG and BSA) have a higher affinity for membrane material, which could be attributed to the fact that these molecules are smaller than casein micelles and, possibly, less hydrophobic [32].

Membrane fouling is more extensive when dairy streams are filtered at higher processing temperatures (45–55 °C) when whey proteins, in particular β-lactoglobulin, are partially unfolded and intensify protein–protein interactions [33]. Moreover, this temperature range facilitates protein–protein and protein–membrane interactions with the participation of divalent cations (Ca^2+^) [32].

### 3.2. Amino Acid Profile

The content of exogenous amino acids and tyrosine in non-fermented milks was influenced by the type of protein preparation (Table 1).

Products with the addition of CN75 and SPC, obtained by membrane filtration of skim milk, were less abundant in threonine, isoleucine and arginine, but more abundant in lysine than commercial casein and whey protein preparations. The type of casein preparation had no effect on the content of leucine, phenylalanine or tyrosine in non-fermented milks. The content of leucine, phenylalanine and tyrosine in non-fermented milks was not affected by the type of whey protein preparation, and leucine content was higher in products enriched with whey protein preparations than casein preparations. In MSPC, changes were found in the content of valine (decrease), isoleucine (decrease) and histidine (increase), relative to the products fortified with commercial whey protein preparations. No differences in the content of valine, isoleucine and histidine were noted in MCN75 and MCN85. A comparison of the products with the addition of protein preparations obtained by membrane filtration and commercial preparations revealed higher concentrations of valine and isoleucine in products fortified with casein preparations, and lower concentrations of leucine, lysine and arginine in products enriched with whey protein preparations. Lower valine and isoleucine levels in milks enriched with SPC than in products fortified with commercial whey protein preparations can probably be attributed to differences in the technology.

The amino acid profile of non-fermented milks was determined by the amino acid composition of major proteins in the added protein preparations as well as the applied method of protein separation. Rennet whey obtained during cheesemaking is used in the production of WPC and WPI. Glycomacro-peptide, a C-terminal part of κ-casein, is released to whey during rennet coagulation, and its content can reach 10–15%. Glycomacro-peptide is an additional source of branched-chain amino acids (isoleucine, leucine and valine) in whey protein preparations obtained from rennet whey. These preparations also contain β-lactoglobulin and α-lactalbumin which are highly abundant in branched-chain amino acids [34].

### 3.3. Calcium and Phosphorus

Non-fermented milks were characterized by a varied content of calcium (*p* < 0.05) and phosphorus (*p* < 0.05), depending on the added protein preparation. Casein preparations increased the calcium content of non-fermented milks relative to MSMP. Milks with the addition of SMP or casein preparations are naturally more abundant in calcium and phosphorus than milks enriched with whey protein preparations. This is because casein micelles contain colloidal calcium phosphate which is transferred to the casein preparation or is retained in SMP. Calcium levels were lowest in MSPC and MWPI (Table 2). According to Misawa et al. [35], 10 mg of whey proteins and casein bound 5.3 mg and 32.7 mg of calcium, respectively. These observations indicate that changes in the percentage share of casein in total protein influence calcium levels in milk.

The content of phosphorus in non-fermented milks decreased after the addition of casein preparations and, to a greater extent, whey proteins. Phosphorus levels were lowest in MSPC. The Ca:P ratio of non-fermented milks enriched with various protein preparations was arranged in the following ascending order: MSMP < MWPI < MCN75 < MCN85 < MSPC < MWPC (Table 2).

Based on the content of calcium and phosphorus in the products, it was calculated how much of them will cover the recommended daily allowance (RDA) of individual groups of consumers. One hundred grams of MCN75 contributed most to meeting RDA of calcium for children aged 4–6, men aged 51–65 and, to a smaller extent, other age groups (Table 3).

A 100 g serving of all non-fermented milks meets the RDA of calcium based on the dietary guidelines for the Polish population in 11.0–17.9% for all age groups [36]. The phosphorus requirements of children aged 4–6 are met in 35.8% by 100 g of MCN75 and in 28.5–33.5% by 100 g of the remaining non-fermented milks. The RDA of phosphorus is covered in 11.4–25.6% in the remaining age groups. The analyzed non-fermented milks are most effective in meeting the calcium and phosphorus requirements of children aged 4–6 because this age group has a lower demand for these nutrients.

### 3.4. Heat Stability and Viscosity

Non-fermented milks enriched with various milk protein preparations were characterized by higher heat stability than milks where the concentration of solids-non-fat increased after the addition of SMP (*p* ≤ 0.05) (Table 4).

The heat stability of non-fermented milks was affected by the type of the added protein preparation. Casein preparations induced a greater increase in heat stability than whey protein preparations. This trend was observed in non-fermented milks enriched with both preparations obtained by membrane filtration and commercial products. The heat stability of non-fermented milks was arranged in the following ascending order depending of the type of added protein preparations: SMP < WPI < SPC, WPC < CN85, CN75 (*p* ≤ 0.05). Active acidity was lowest in the control sample (6.57). Non-fermented milks with the addition of SMP, CN75 and CN85 had a pH of 6.60. The pH of non-fermented milks enriched with WPI and WPC ranged from 6.62 to 6.63, whereas the highest pH was noted in milk with the addition of SPC (6.67). The heat stability of non-fermented milks is not bound by a simple linear relationship with their pH, but it is influenced by protein type, calcium and phosphorus content, and the Ca:P ratio. Despite the fact that whey proteins are less thermally stable than casein [14], milks enriched with SPC, WPC and WPI were characterized by high heat stability, probably due to a high pH, and in products fortified with SPC and WPI, due to lower calcium content relative to the control sample or samples enriched with casein preparations.

A mathematical analysis of the flow curves of all non-fermented milks revealed a linear relationship between shear stress and shear rate, with a coefficient of determination of R^2^ > 0.99. The flow curve analysis also demonstrated that the analyzed samples exhibited the properties of Newtonian fluids. The viscosity of non-fermented milks varied subject to the applied additive (*p* ≤ 0.05) and was arranged in the following ascending order: CN85 < CN75, WPC < SPC < WPI. Milk proteins, including whey proteins, bind water, affect the rheological properties of dairy products, and may contribute to increasing their viscosity [13].

### 3.5. Color

Color parameters L*, a* and b* and the calculated color saturation and total color difference in non-fermented milks enriched with protein preparations differed significantly from the control sample (*p* ≤ 0.05) (Table 5).

The value of parameter L* was highest in milk with the addition of protein preparations CN75 and SPC, obtained by membrane filtration. The remaining non-fermented milks with the addition of commercial protein preparations were characterized by lower values of L*. Parameters a* (greenness/redness) and b* (blueness/yellowness) were highest in MWPI (Table 5). High values of L*, a* and b* in non-fermented milks with the addition of CN75 and SPC contributed to color saturation and total color difference against a white standard. Samples MCN75 and MWPC did not differ in any of the analyzed color parameters and formed homogeneous groups. MCN75 and MWPC were characterized by higher (*p* ≤ 0.05) color saturation and higher total color difference than milks enriched with the remaining protein preparations and SM. According to Cheng et al. [37], parameter L* is influenced by protein content and protein composition, i.e., the higher the total protein content and the higher the casein’s share of total protein in low-fat milk, the higher the value of L*. High casein content exerts a greater influence on the values of L* (increase), a* (increase) and b* (decrease) than total protein content [35]. Parameters a* and b* are affected by factors associated with the natural content of milk pigments (content of carotenoids, protein and riboflavin) [38]. However, the analyzed non-fermented milks were produced from skim milk; therefore, the observed differences in color parameters probably did not result from variations in the content of carotenoids as fat-soluble components. Unlike carotenoids, riboflavin is soluble in water. In a study by Magan et al. [39], riboflavin content was 5–8 times higher in dried whey products than in SMP.

The type of protein preparation applied in the production process can induce differences in the color of non-fermented milks. According to Milovanovic et al. [40], in milk powders, parameters L*, a* and b* are determined by milk source, chemical composition of milk powders (content and type of protein), the applied drying method and drying conditions. Most protein powders have a white-gray color. In stored products, color darkening is caused by non-enzymatic browning reactions, in particular in powders with high concentrations of lactose and proteins abundant in lysine. Lactose may undergo crystallization during the production or storage of milk powders, which induces a local increase in the concentration of mineral salts and contributes to denaturation of milk proteins [41].

### 3.6. Sensory Analysis

Five main attributes were evaluated in the sensory analysis of non-fermented milks fortified with protein preparations: appearance, aroma, consistency, mouthfeel and flavor. The results are presented in Table 6.

An assessment of the products’ appearance revealed that all tested non-fermented milks were characterized by highly uniform color distribution, without visible separation of layers or adhesion to packaging walls. Gray and beige discoloration was not observed, and the average values of these attributes (1.8 to 2.0) were indicative of white color. This is a highly satisfactory result which indicates that none of the tested products had a white-gray color which is typical of milk protein concentrates [13] and is unacceptable for consumers. However, significant differences in creamy color were noted between the evaluated products. Samples MSMP and MCN75 were evaluated as white, whereas the remaining samples were regarded as moderately creamy in color. Creamy color was most apparent in non-fermented milks with the addition of milk serum proteins (MSPC) and whey protein isolate (MWPI). These observations could be attributed to the fact that the presence of riboflavin (vitamin B2) contributes to the yellow color of whey [5,42].

In the sensory analysis of aroma, non-fermented milks were assessed for the overall aroma intensity, perceptions of milky aroma, milk powder aroma, sweet aroma and whey aroma. The overall aroma intensity was most discernible in MWPC; it was moderate in the remaining samples, and least detectable in milks with the addition of both casein preparations. A similar observation was made in the evaluation of milky aroma, where products MCN75 and MCN85 received the lowest mean scores. In turn, the milk powder aroma was strongly associated with the sweet aroma.

The panelists were familiarized with the sensory attributes of the analyzed protein preparations before the assessment. As a result, they were able to identify the intensity of the aromas characteristic of the tested additives, and described them as weakly detectable in all samples. The intensity of foreign odors was determined as weak. The addition of SMP and the remaining preparations produced a weakly detectable pasteurization aroma. Buttery, sour and metallic off-odors were not identified.

The consistency of the studied non-fermented milks was evaluated as highly uniform, watery and smooth (devoid of grains). Viscosity was somewhat higher in MSPC, MWPI and MWPC, but the observed differences were not significant (*p* > 0.05).

Smoothness received the highest scores in the evaluation of mouthfeel, in particular in MSPC, MWPI and MWPC (Table 6). Notably, this parameter received a somewhat lower score in milk with the highest casein content (MCN85), which contributed to a moderate mealy mouthfeel in this product. All of the tested products were characterized by low fatty mouthfeel, and the results of the statistical analysis demonstrated that the fatty sensation was more pronounced in MWPI, MWPC and MCN85 (*p* = 0.005). Sticky mouthfeel was evaluated as negligible in all products, and product thickness was described as watery and characteristic of milk.

In the taste analysis, non-fermented milks differed significantly in four out of the thirteen evaluated sensory parameters. The differentiating attributes were: flavor typical of a given additive, milk powder flavor, sweet flavor, and whey flavor. A flavor typical of a given additive was not detected in MSMP or MCN85, and it was strongest (moderate score) in MWPC. The above product was also characterized by the most intense milk powder flavor and, together with MWPI, by the highest sweetness. The sweet flavor of milks containing WPI and WPC could be attributed to the high lactose content of whey [43] used in the production of whey protein concentrate and isolate, as well as the transfer of lactose to the final product [7]. The sweet flavor of non-fermented milks was also confirmed by Królczyk et al. [44], who used high-protein whey preparations as substitutes for sucrose and artificial sweeteners. The whey flavor was moderately detectable in milks fortified with whey protein concentrate and isolate. Unlike in the study by Evans et al. [12], the analyzed products did not have a cardboard off-flavor. Equally importantly, salty, sour, bitter, chalky and astringent flavors were not identified in any of the tested products.

## 4. Conclusions

The application of the whey protein preparation increased the proportion of whey proteins in the total protein content of non-fermented milks, whereas the addition of casein did not induce changes in the ratio of casein to whey proteins. All of the tested protein preparations decreased the phosphorus content of non-fermented milks, and the addition of serum proteins and the whey protein isolate additionally decreased the content of calcium relative to the product with the addition of SMP. Non-fermented milks enriched with casein preparations, in particular those obtained by membrane filtration, contained more calcium than the control sample. In children aged 4–7, a 100 g serving of non-fermented milk with the addition of CN75 meets the RDA of calcium and phosphorus in 17.9% and 36.8%, respectively, whereas other products fortified with milk proteins are less effective in covering the calcium and phosphorus requirements of children. In the remaining age groups, the demand for mineral salts was met to a lesser extent by a 100 g serving of this milk. In the group of the analyzed exogenous amino acids, the addition of casein and milk serum proteins obtained by membrane filtration clearly increased the lysine content of non-fermented milks.

Non-fermented milks enriched with milk proteins, in particular casein, were characterized by higher heat stability than milk with the addition of SMP. These results indicate that the analyzed protein preparations are useful in the production of thermally processed dairy foods. Non-fermented milks were also characterized by satisfactory sensory attributes. The milk enriched with whey protein isolate, followed by the milk fortified with milk serum protein concentrate, received the highest scores in the sensory evaluation. The product with the addition of serum whey concentrate was characterized by a too strong milk powder flavor. In turn, casein preparations did not impart an intensive aroma to the fortified products, and CN85 additionally contributed to a mealy mouthfeel. Non-fermented milks with the addition of protein preparations obtained by membrane filtration of skim milk have comparable sensory attributes to non-fermented milks enriched with commercial protein preparations. An analysis of the nutritional value, technological properties and sensory attributes of protein-fortified non-fermented milks indicates that the designed milks offer a good alternative to products targeting consumers with specific nutritional needs and dietary preferences.

The presented results make a valuable contribution to the production of plain or flavored non-fermented milks enriched with various protein fractions. The study proposes a ready-to-use technology for the production of protein preparations and non-fermented milks enhanced with protein preparations, which can be implemented in industrial dairy plants.

## Figures and Tables

**Table 1:** Amino acid profile of non-fermented milks enriched with protein preparations, g/100 g of amino acids.

Amino Acid	MSMP	MCN75	MCN85	MSPC	MWPI	MWPC
Threonine	8.33 ± 0.16 ^cd^	6.84 ± 0.55 ^b^	8.75 ± 0.01 ^cd^	4.81 ± 0.04 ^a^	7.93 ± 0.12 ^c^	8.98 ± 0.30 ^d^
Valine	11.96 ± 0.08 ^bc^	11.61 ± 0.28 ^bc^	12.43 ± 0.02 ^c^	10.03 ± 0.45 ^a^	11.30 ± 0.18 ^b^	11.45 ± 0.24 ^b^
Isoleucine	9.98 ± 0.07 ^bc^	9.47 ± 0.21 ^ab^	12.57 ± 0.01 ^c^	9.04 ± 0.51 ^a^	10.22 ± 0.13 ^bc^	10.06 ± 0.02 ^bc^
Leucine	24.27 ± 0.08 ^a^	24.40 ± 0.14 ^a^	23.92 ± 0.01 ^a^	26.08 ± 0.21 ^bc^	27.25 ± 0.53 ^c^	25.81 ± 0.63 ^b^
Phenylalanine	8.33 ± 0.15 ^b^	7.90 ± 0.04 ^ab^	8.18 ± 0.02 ^b^	7.17 ± 0.47 ^a^	7.16 ± 0.36 ^a^	7.56 ± 0.08 ^ab^
Histidine	4. 96 ± 0.01 ^ab^	5.27 ± 0.07 ^cd^	5.49 ± 0.01 ^d^	5.10 ± 0.12 ^bc^	4.82 ± 0.08 ^ab^	4.79 ± 0.13 ^a^
Lysine	16.88 ± 0.49 ^ab^	20.09 ± 0.99 ^b^	14.93 ± 0.02 ^a^	25.10±2.42 ^c^	17.85 ± 0.07 ^ab^	16.45 ± 0.13 ^ab^
Arginine	5.91 ± 0.04 ^bc^	5.76 ± 0.16 ^b^	6.85 ± 0.01 ^d^	5.32 ± 0.05 ^a^	5.78 ± 0.09 ^b^	6.09 ± 0.12 ^c^
Tyrosine	9.38 ± 0.20 ^b^	8.66 ± 0.44 ^ab^	9.13 ± 0.01 ^b^	7.35 ± 0.81 ^a^	7.71 ± 0.85 ^ab^	8.81 ± 0.12 ^ab^

Values are means ± SD (*n* = 2). Values with different superscripts in rows differ significantly at *p* ≤ 0.05. MSMP—milk with skim milk powder, control sample; MCN75—milk with micellar casein concentrate with 75% protein content; MCN85—milk with micellar casein concentrate with 85% protein content; MSPC—milk with serum protein; MWPI—milk with whey protein isolate; MWPC—milk with whey protein concentrate.

**Table 2:** Content of calcium and phosphorus and the Ca:P ratio in non-fermented milks enriched with protein preparations.

Sample	Calcium, mg 100 g^−1^	Phosphorus, mg 100 g^−1^	Ca:P Ratio
MSMP	164.24 ± 0.41 ^c^	112.81 ± 0.36 ^f^	1.46 ± 0.02 ^a^
MCN75	178.85 ± 0.58 ^f^	90.63 ± 0.38 ^e^	1.97 ± 0.01 ^c^
MCN85	166.60 ± 0.14 ^d^	83.11 ± 1.05 ^d^	2.00 ± 0.03 ^d^
MSPC	142.38 ± 0.17 ^a^	66.32 ± 0.24 ^a^	2.15 ± 0.01 ^e^
MWPI	149.85 ± 0.43 ^b^	79.72 ± 0.25 ^c^	1.88 ± 0.01 ^b^
MWPC	167.54 ± 0.50 ^e^	72.08 ± 0.24 ^b^	2.32 ± 0.01 ^f^

Values are means ± SD (*n* = 2). Values with different superscripts in rows differ significantly at *p* ≤ 0.05. MSMP—milk with skim milk powder, control sample; MCN75—milk with micellar casein concentrate with 75% protein content; MCN85—milk with micellar casein concentrate with 85% protein content; MSPC—milk with serum protein; MWPI—milk with whey protein isolate; MWPC—milk with whey protein concentrate.

**Table 3:** Daily nutrient intake per 100 g of non-fermented milks enriched with protein preparations (%).

Mineral	Sex/Age (years) Group	RDA * (mg/day)	MSMP	MCN75	MCN85	MSPC	MWPI	MWPC
Calcium	Children aged 4–6	1000	16.4	17.9	16.7	14.2	15.0	16.8
	Boys aged 13–15	1300	12.6	13.8	12.8	11.0	11.5	12.9
	Men aged 51–65	1000	16.4	17.9	16.7	14.2	15.0	16.8
	Girls aged 13–15	1300	12.6	13.8	12.8	11.0	11.5	12.9
	Women aged 51–65	1200	13.7	14.9	13,9	11.9	12.5	14.0
Phosphorus	Children aged 4–6	500	32.8	35.8	33.3	28.5	30.0	33.5
	Boys aged 13–15	1250	13.1	14.3	13.3	11.4	12.0	13.4
	Men aged 51–65	700	23.5	25.6	23.8	20.3	21.4	23.9
	Girls aged 13–15	1250	13.1	14.3	13.3	11.4	12.0	13.4
	Women aged 51–65	700	23.5	25.6	23.8	20.3	21.4	23.9

Calculations of results was based on Wojtasik et al. [36] * RDA—Recommended Dietary Allowance based on the dietary guidelines for the Polish population; MSMP—milk with skim milk powder, control sample; MCN75—milk with micellar casein concentrate with 75% protein content; MCN85—milk with micellar casein concentrate with 85% protein content; MSPC—milk with serum protein; MWPI—milk with whey protein isolate; MWPC—milk with whey protein concentrate.

**Table 4:** Viscosity and heat stability of non-fermented milks enriched with protein preparations.

Sample	Viscosity mPa s	Heat Stability min
MSMP	2.8 ± 0.1 ^a^	5.32 ± 0.08 ^a^
MCN75	3.3 ± 0.1 ^b^	8.42 ± 0.03 ^c^
MCN85	3.2 ± 0.1 ^b^	8.40 ± 0.13 ^c^
MSPC	4.1 ± 0.1 ^c^	7.49 ± 0.22 ^b^
MWPI	4.8 ± 0.1 ^d^	7.51 ± 0.12 ^b^
MWPC	3.2 ± 0.1 ^b^	7.53 ± 0.20 ^b^

Values are means ± SD (*n* = 2). Values with different superscripts in rows differs significantly at *p* ≤ 0.05. MSMP—milk with skim milk powder, control sample; MCN75—milk with micellar casein concentrate with 75% protein content; MCN85—milk with micellar casein concentrate with 85% protein content; MSPC—milk with serum protein; MWPI—milk with whey protein isolate; MWPC—milk with whey protein concentrate.

**Table 5:** Color parameters of non-fermented milks enriched with protein preparations.

Sample	L*	a*	b*	C*	ΔE
MSMP	81.51 ± 0.13 ^a^	−5.84 ± 0.01 ^a^	0.87 ± 0.08 ^b^	5.91 ± 0.02 ^b^	49.76 ± 0.03 ^b^
MCN75	82.83 ± 0.13 ^c^	−5.67 ± 0.01 ^c^	2.79 ± 0.04 ^c^	6.32 ± 0.02 ^d^	54.23 ± 0.13 ^e^
MCN85	82.49± 0.08 ^b^	−5.71 ± 0.02 ^b^	0.36 ± 0.01 ^a^	5.73 ± 0.01 ^a^	46.59 ± 0.13 ^a^
MSPC	83.18 ± 0.14 ^d^	−5.03 ± 0.01 ^d^	3.63 ± 0.06 ^d^	6.20 ± 0.04 ^c^	52.64 ± 0.22 ^d^
MWPI	82.46 ± 0.06 ^b^	−4.03 ± 0.02 ^e^	4.35 ± 0.03 ^e^	5.92 ± 0.03 ^b^	50.41 ± 0.33 ^c^
MWPC	82.76 ± 0.24 ^c^	−5.65 ± 0.02 ^c^	2.77 ± 0.05 ^c^	6.29 ± 0.03 ^d^	53.92 ± 0.23 ^e^

Values are means ± SD (*n* = 2). Values with different superscripts in rows differ significantly at *p* ≤ 0.05. C*—color saturation; ΔE—total difference in color; MSMP—milk with skim milk powder, control sample; MCN75—milk with micellar casein concentrate with 75% protein content; MCN85—milk with micellar casein concentrate with 85% protein content; MSPC—milk with serum protein; MWPI—milk with whey protein isolate; MWPC—milk with whey protein concentrate.

**Table 6:** Mean sensory scores of non-fermented milks enriched with protein preparations.

	SAMPLE	MSMP	MCN75	MCN85	MSPC	MWPI	MWPC	*p*-Value
APPEARANCE	Creamy color	2.0 ^a^	2.0 ^a^	2.4 ^ab^	2.8 ^b^	3.0 ^b^	2.6 ^ab^	0.014
	Beige color	1.8	1.8	1.8	1.8	1.8	1.8	>0.05
	Gray color	1.9	1.9	1.8	1.9	2.0	1.9	>0.05
	Uniform color distribution	5.0	5.0	5.0	5.0	5.0	5.0	>0.05
	Layer separation	1.0	1.0	1.0	1.0	1.0	1.0	>0.05
	Adhesion to packaging	1.0	1.0	1.0	1.0	1.0	1.0	>0.05
AROMA	Overall intensity	2.6 ^abc^	2.5 ^ab^	2.3 ^a^	3.0 ^cd^	2.9 ^bcd^	3.3 ^d^	0.000
	Typical of additive	1.3	1.3	1.3	1.4	1.3	1.4	>0.05
	Milky	2.6 ^ab^	2.3 ^a^	2.3 ^a^	2.9 ^b^	2.9 ^b^	2.9 ^b^	0.003
	Buttery	1.0	1.0	1.0	1.0	1.0	1.0	>0.05
	Milk powder	1.4 ^a^	1.4 ^a^	1.5 ^a^	2.6 ^b^	2.6 ^b^	2.6 ^b^	0.000
	Sweet	1.5 ^a^	1.5 ^a^	1.5 ^a^	2.0 ^ab^	2.3 ^b^	2.3 ^b^	0.016
	Sour	1.0	1.0	1.0	1.0	1.0	1.0	>0.05
	Whey	1.0 ^a^	1.1 ^a^	1.1 ^a^	1.1 ^a^	1.9 ^b^	1.9 ^b^	0.000
	Pasteurization	1.5	1.5	1.5	1.5	1.5	1.5	>0.05
	Metallic	1.0	1.0	1.0	1.0	1.0	1.0	>0.05
	Foreign	1.1	1.4	1.3	1.3	1.3	1.3	>0.05
CONSISTENCY	Homogeneous	4.9	4.9	4.9	4.9	4.9	4.9	>0.05
	Watery	3.5	3.5	3.5	3.5	3.5	3.5	>0.05
	Adhesive	1.0	1.0	1.0	1.3	1.3	1.3	>0.05
	Grainy	1.0	1.0	1.0	1.0	1.0	1.0	>0.05
	Smooth mouthfeel	4.0	4.0	3.9	4.3	4.3	4.3	>0.05
	Mealy mouthfeel	1.4 ^a^	1.4 ^a^	2.1 ^b^	1.3 ^a^	1.3 ^a^	1.3 ^a^	0.021
	Fatty mouthfeel	1.4 ^a^	1.5 ^ab^	2.0 ^c^	1.5 ^ab^	1.9 ^bc^	2.0 ^c^	0.005
	Sticky mouthfeel	1.1	1.1	1.1	1.1	1.1	1.1	>0.05
	Thickness	1.0	1.0	1.3	1.3	1.3	1.3	>0.05
TASTE	Milky	2.1	2.0	2.0	2.0	2.1	2.0	>0.05
	Typical of additive	1.0 ^a^	1.1 ^a^	1.0 ^a^	1.9 ^b^	1.9 ^b^	2.3 ^c^	0.000
	Milk powder	2.8 ^ab^	2.3 ^a^	2.4 ^a^	3.1 ^b^	3.0 ^b^	3.9 ^c^	0.000
	Sweet	3.4 ^b^	2.5 ^a^	2.5 ^a^	2.9 ^ab^	3.6 ^b^	3.6 ^b^	0.002
	Salty	1.3	1.1	1.1	1.1	1.1	1.1	>0.05
	Sour	1.0	1.0	1.0	1.0	1.0	1.0	>0.05
	Bitter	1.0	1.0	1.0	1.0	1.0	1.0	>0.05
	Whey	1.1 ^a^	1.3 ^a^	1.3 ^a^	1.4 ^a^	2.4 ^b^	2.5 ^b^	0.004
	Chalky	1.0	1.0	1.0	1.0	1.0	1.1	>0.05
	Cardboard	1.0	1.0	1.1	1.0	1.0	1.1	>0.05
	Astringent	1.0	1.0	1.0	1.0	1.0	1.0	>0.05
	Pasteurization	1.6	1.6	1.5	1.6	1.6	1.6	>0.05
	Foreign	1.4	1.4	1.3	1.3	1.3	1.4	>0.05

Values are means ± SD (*n* = 8). Values with different superscripts in rows differ significantly at *p* ≤ 0.05. MSMP—milk with skim milk powder, control sample; MCN75—milk with micellar casein concentrate with 75% protein content; MCN85—milk with micellar casein concentrate with 85% protein content; MSPC—milk with serum protein; MWPI—milk with whey protein isolate; MWPC—milk with whey protein concentrate.

## Data Availability

The datasets generated for this study are available on request to the corresponding author.
